# Icariin Inhibits Pulmonary Hypertension Induced by Monocrotaline through Enhancement of NO/cGMP Signaling Pathway in Rats

**DOI:** 10.1155/2016/7915415

**Published:** 2016-05-30

**Authors:** Li-sheng Li, Yun-mei Luo, Juan Liu, Yu Zhang, Xiao-xia Fu, Dan-li Yang

**Affiliations:** ^1^Department of Pharmacology, Key Lab of Basic Pharmacology of Education Ministry, Zunyi Medical College, No. 201 Dalian Road, Zunyi, Guizhou 563099, China; ^2^Institute of Clinical Medicine, Affiliated Hospital of Zunyi Medical College, No. 149 Dalian Road, Zunyi, Guizhou 563099, China

## Abstract

It has been reported that icariin (ICA) increased contents of nitric oxide (NO) and cyclic guanosine monophosphate (cGMP) by improving expression of endothelial nitric oxide synthase (eNOS) and inhibition of phosphodiesterase type 5 (PDE5). In addition, dysfunction of the NO/cGMP pathway may play a crucial role in the pathogenesis of pulmonary hypertension (PH). In this study, the potential protective effects of ICA on PH induced by monocrotaline (MCT, 50 mg/kg) singly subcutaneous injection were investigated and the possible mechanisms involved in NO/cGMP pathway were explored in male Sprague Dawley rats. The results showed that ICA (20, 40, and 80 mg/kg/d) treatment by intragastric administration could significantly ameliorate PH and upregulate the expression of eNOS gene and downregulate the expression of PDE5 gene in MCT-treated rats. Both ICA (40 mg/kg/d) and L-arginine (200 mg/kg/d), a precursor of NO as positive control, notably increased the contents of NO and cGMP in lung tissue homogenate, which were inversed by treatment with ^*N*^G-nitro-L-arginine-methyl ester (L-NAME), a NOS inhibitor, and L-NAME-treatment could also inhibit the protective effects of ICA (40 mg/kg/d) on mean pulmonary artery pressure and artery remodeling and tends to inhibit right ventricle hypertrophy index. In summary, ICA is effective in protecting against MCT-induced PH in rats through enhancement of NO/cGMP signaling pathway in rats.

## 1. Introduction

Pulmonary hypertension (PH) is a chronic progressive and devastating disease in which mean pulmonary arterial pressure (mPAP) increases by more than 25 mmHg in the resting state and finally leads to right ventricular failure [1]. The disease is characterized by the difficulty in determining its origination and diagnosis, poor prognosis, and a high mortality rate (about 15% annually) [2]. Although the exact pathogenesis of PH is not fully understood, the considerable evidence from PH animal experiments and patients suggest nitric oxide/cyclic guanosine monophosphate (NO/cGMP) signaling pathway dysfunction is a key event in the PH pathophysiology process [3, 4], which leads to vasoconstriction and arterial remodeling of small pulmonary arteries. The vicious cycle being formed between arterial remodeling and elevation of pulmonary arterial obstruction promotes gradually development of the disease. So it is helpful towards the treatment of PH to investigate any reagents that ameliorate vascular remodeling and/or decrease pulmonary arterial obstruction [5]. On the other hand, recent studies in animal model of PH induced by monocrotaline (MCT) show unchanged NO content and endothelial nitric oxide synthase (eNOS) expression in the early MCT treatment [6]; Sawamura et al. found also there was no significant change in pulmonary cGMP levels 3 weeks after MCT (60 mg/kg) injection [7], but it is worth to be sure that to enhance function of NO/cGMP signaling pathway can improve PH. The function of NO/cGMP signaling pathway is modulated by three key enzymes in the cardiovascular system, namely, eNOS, soluble guanylate cyclase (sGC), and phosphodiesterase type 5 (PDE5) [8–10]. NO in blood serum is mainly produced by eNOS through the conversion of L-arginine (L-arg) into L-citrulline in vascular endothelial cells, and NO in turn activates sGC; the latter promotes conversion of guanosine triphosphate (GTP) to cGMP [11]. cGMP further activates protein kinase G and subsequently develops the wide range of bioactivities, including vascular relaxation and inhibition of vascular smooth cell proliferation in the cardiovascular system [12–14]. PDE5 can terminate the action of cGMP by improving hydrolysis of cGMP, which is expressed at a higher level in the pulmonary circulation than in systemic vessels [15]. The presented facts clearly demonstrated that NO/cGMP signaling pathway dysfunction is involved in the pathogenesis of PH and also is an important target for the development of new drugs such as PDE5 inhibitor sildenafil or soluble guanylate cyclase (sGC) stimulator BAY 63-252.

Icariin (ICA, PubCHem CID: 5318997), a major compound of* herb epimedium*, is a well-known and popular traditional Chinese medicine to treat erectile dysfunction. At present, abundant pharmacological functions of ICA have been identified including anti-inflammation, antioxidative stress reduction, anticancer, cardiovascular protection, stimulation of osteoblast proliferation, and enhanced immune function [16, 17]. Recently, ICA has been demonstrated to act as a PDE5 inhibitor with IC_50_ values of 1.0, 0.75, and 1.1 *μ*M, respectively, an inhibitor of PDE5A1, A2, and A3, and to increase cGMP concentration in cavernous smooth muscle cells* in vitro* [18, 19], and it can also notably upregulate eNOS expression in porcine aorta endothelial cells [20]. On the basis of these facts, we hypothesize that ICA may function as anti-PH. The present study was designed to investigate (1) the potential effect of ICA on PH induced by subcutaneous injection MCT in rats and (2) the relationship between the possible anti-PH effect and NO/cGMP signaling pathway.

## 2. Materials and Methods

### 2.1. Animals and Reagents

Adult male Sprague-Dawley rats (*n* = 108, weight 200 to 250 g, SPF) were purchased from the Experiment Animal Center of Institute of Surgery Research of the Third Military Medical University (Chongqing, China) and were housed in SPF-grade animal facilities (Certificate Number: SYXK 2011-004) of Zunyi Medical College (Guizhou, China). Animals were allowed free access to a standard laboratory rat diet and water ad libitum. ICA (purity: 98%) was obtained from Nanjing Zelang Medical Technology Co., Ltd. (Nanjing, China). Sildenafil (SIL) was product of Pfizer Inc. (New York, USA). Monocrotaline (MCT), L-arg, and NG-nitro-L-arginine-methyl ester (L-NAME) were bought from Sigma-Aldrich Co. (St. Louis, MO, USA). The study protocol was approved by the ethics committee of Zunyi Medical College.

### 2.2. PH Model and Experimental Protocol

The rats were given single subcutaneous injection with either MCT (50 mg/kg) or equal vehicle as a control after they were acclimatized for 1 week [21]. Then, all rats were randomly divided into 8 groups with 12 rats allocated to each group as follows: control, model, ICA (20, 40, and 80 mg/kg/d, intragastric administration), and SIL (25 mg/kg/d, intragastric administration). For analyzing whether the effects of ICA on PH model were related to NO/cGMP signaling pathway, two groups were given L-NAME (20 mg/kg/d, intragastric administration) combined with ICA (40 mg/kg/d, intragastric administration) or L-arg (200 mg/kg/d, intraperitoneal injection), respectively. All animals received equal volume of vehicle for different reagents. On the 8th day after MCT injection, the animals were treated according to the above protocol for 3 consecutive weeks. All rats were weighed every 2 days to enable dose adjustment during this period.

### 2.3. mPAP Measurement

All surviving rats were anesthetized with pentobarbital sodium (50 mg/kg, intraperitoneal injection). For assessment of mPAP, a central venous catheter (Secalon, 16 G/1.6 × 400 mm, Viggo pruducts, Swindon, UK) was bent about 90 degrees with the help of a thin metallic wire and took shape in 60°C water for 10 min at tip of catheter in advance. The catheter connected to a pressure transducer was inserted firstly into the right subclavian vein at 0.5 cm overhead clavicle and advanced into the RA, RV, and PA. Whether the catheter had reached the pulmonary artery was judged on the basis of changes of the pressure curve shown on a monitor connected to a Powerlab system (ADInstruments, Sydney, Australia).

### 2.4. Surgical Operation and Tissue Harvest

After the final hemodynamic assessment, arterial blood (0.5 mL) was collected from another catheter placed in right carotid artery and isolated completely from air for measurement of partial pressure of oxygen and carbon dioxide by Cobas b123 type Blood Gas Analyzer (Roche, Basel, Switzerland). Then, all animals were euthanized by exsanguination. The heart was removed quickly, and RV and the left ventricle with septum were separated and weighed separately, and the right ventricle hypertrophy index (RVHI) for each rat was calculated using the following formula: RV weight/(LV + septum) weight × 100%. The right lung tissue and pulmonary artery were together separated, the lung tissue was subsequently perfused with 30 mmHg perfusion pressure by pulmonary artery with 10% buffered formalin until without blood in lavage fluid, and then the right lower pulmonary lobe was fixed in 10% buffered formalin for histological analysis. The left lungs were separated, washed with cold physiological salt solution, and kept in liquid nitrogen for biochemical analysis.

### 2.5. Measurement of cGMP and NO Level in Lung Tissue

Lung tissues separated for cGMP and NO level assay were put into 1 mL PBS buffer solution (137 mM NaCl, 2.7 mM KCl, 4.3 mM NaH_2_PO_4_, and 1.4 mM K_2_HPO_4_, pH 7.4) and homogenized using a manual tissue homogenizer and then centrifuged (3000 rpm, 4°C, 10 min). The supernatant was collected, and the protein concentrations in supernatant were detected using the BCA protein assay Kit (Beyotime, Shanghai, China). The contents of the target substance per milligram of protein were standardized in accordance with the protein concentration of corresponding samples. The levels of cGMP were detected using Enzyme-linked Immunosorbent Assay Kits (R&D System, Minneapolis, USA). NO is rapidly oxidized to nitrite and nitrate which are used to quantitate NO production, and nitrite and nitrate levels were assayed by a Nitric Oxide Colorimetric Assay Kit (Biovision, Milpitas, USA) according to the manufacturer's instruction. In the text nitrite and nitrate levels were still expressed in NO.

### 2.6. Western Blot Assay

The frozen lung tissue samples conserved in liquid nitrogen were cut into pieces and put into 1 mL RIPA lysing buffer supplement with 1 nM PMSF (Beyotime, Shanghai, China). The samples were homogenized using a manual tissue homogenizer and then centrifuged (12000 ×g, 4°C, 10 min) and the supernatants were collected. Total protein in supernatants was quantified by BCA protein assay Kit (Beyotime, Shanghai, China) and subjected to Western blot analysis. Each sample (containing protein 100 *μ*g) was hyperthermally denatured at 95°C for 5 min and electrophoretically separated on 5 to 10% gradient SDS-PAGE gels and transferred to a PVDF (0.45 *μ*m) membrane. The membranes were blocked with 5% defatted milk in PBS buffer for 2 h at room temperature and then incubated, respectively, with anti-PDE5 polyclonal antibody (1 : 500, Abcam, Cambridge, USA), anti-eNOS polyclonal antibody (1 : 1000, Abcam, Cambridge, USA), and anti-*β*-actin monoclonal antibody (1 : 2000, Abcam, Cambridge, USA) at 4°C overnight, followed by incubation with an appropriate horseradish peroxidase conjugated secondary antibody at room temperature for 2 h with gentle rotation. The membranes were visualized using chemiluminescence reagent BeyoECL Plus (Beyotime, Shanghai, China). The image was scanned and band densities were quantified using Quantity One 1D analysis software v4.52 (BioRad, Hercules, USA). *β*-actin was used to normalize protein loading.

### 2.7. RNA Isolation and Real-Time RT-PCR

Total RNA was isolated from lung tissue using Trizol*™* (Beyotime, Shanghai, China) and purified by RNeasy mini kit (Qiagen Co., Valencia, USA). Reverse transcription of total RNA was performed with MuLV reverse transcriptase and OligodT primer. iCycler iQ Real-Time PCR Detection System (BIO-RAD Co., CA, USA) was employed to execute Real-Time Polymerase Chain Reaction (PCR) with SYBR® Green PCR Master Mix (ABI Co., Foster, USA). The primers were designed and synthesized by TaKaRa Biological Engineering Com (TaKaRa, Dalian, China). The following primers were used: eNOS (GenBank Acc. NM_021838.2) forward, 5-CAA GAC CGA TTA CAC GAC ATT GAGA-3, reverse, 5-TGA GGA CTT GTC CAA ACA CTC CAC-3 (148 bp product); PDE5 (GenBank Acc. NM_133584.1) forward, 5-AAT TGG AGG CAC GCC TTT AACA-3; reverse, 5-TCA TGG CTT AAA GCG GCA ATC-3 (122 bp product); *β*-actin (GenBank Acc. NM_031144.2 ) forward, 5-GGA GAT TAC TGC CCT GGC TCC TA-3; reverse, 5-GAC TCA TCG TAC TCC TGC TTG CTG-3 (150 bp product). The reactions conditions were as follows: (1) 95°C 8 min 1 cycle; (2) 95°C 15 s 60°C 1 min 40 cycles. The results (Ct values) of target gene were normalized with *β*-actin of the same sample and expressed relative to controls.

### 2.8. Histomorphology Assay

Lung tissues from the same location (marginal right lower pulmonary lobes) were harvested from the surviving rats and immersed fully and fixed with 4% paraformaldehyde solution for 8 h and then embedded in paraffin wax. Tissue blocks were sectioned to 5 *μ*m in thickness and stained with hematoxylin & eosin (H&E) according to common histopathological procedures. Pulmonary artery remodeling was assessed by measuring the area of vessels wall (diameter 50 to 100 *μ*m) [17]. Arterial wall and section areas were measured by an observer, who was blinded to the treatments of the rats; the measurements were conducted under 200x magnification with a microscope (Leica, Wetzlar, Germany) and computerized morphometric system with the software LAS 3.8 (Leica microsystem, Heerbrugg, Switzerland). The percentage of arterial wall area was calculated according to the following formula: arterial wall area/arterial section area × 100% [18].

### 2.9. Statistical Analysis

All data were presented as mean ± SD and analyzed statistically using the SPSS 19.0 software (SPSS Inc., Chicago, USA). Survival curves were derived by the Kaplan-Meier method and compared by Log-Rank tests. The normality of other data was analyzed statistically by the K-S test. The normal-distributed data firstly were analyzed statistically via one-way analysis of variance (ANOVA), and the statistical significance of difference between two groups was determined using LSD method if equal variance or Dunnett's T3 method if missing variance. Statistical significance was accepted at *p* < 0.05.

## 3. Results

### 3.1. ICA Administration Enhances Survival Rates of MCT-PH Model Rats

Just 10 days after MCT injection, rats started to die in model group and ICA20 group; subsequently rats in all groups intermittently died except control group. The survival rate of model group was 42% at the 28th day after MCT treatment, which was notably lower than control group (100%) (*p* < 0.05); the survival rates in ICA and L-arg treatment groups increase (they were 58.3%, 75%, 75%, and 66.7% in ICA20, ICA40, ICA80, and L-arg groups, resp.). The survival rate of L-arg (200 mg/kg/d) with L-NAME (20 mg/kg/d) group and ICA (40 mg/kg/d) with L-NAME (20 mg/kg/d) group was, respectively, 66.7% and 58.3%, but there was no statistical difference in comparison with that in the ICA40 group and the L-arg group (*p* > 0.05).

### 3.2. ICA Treatment Decreases mPAP and Ameliorates Right Ventricle Hypertrophy in MCT-PH Model Rats

**Figure 1 fig1:**
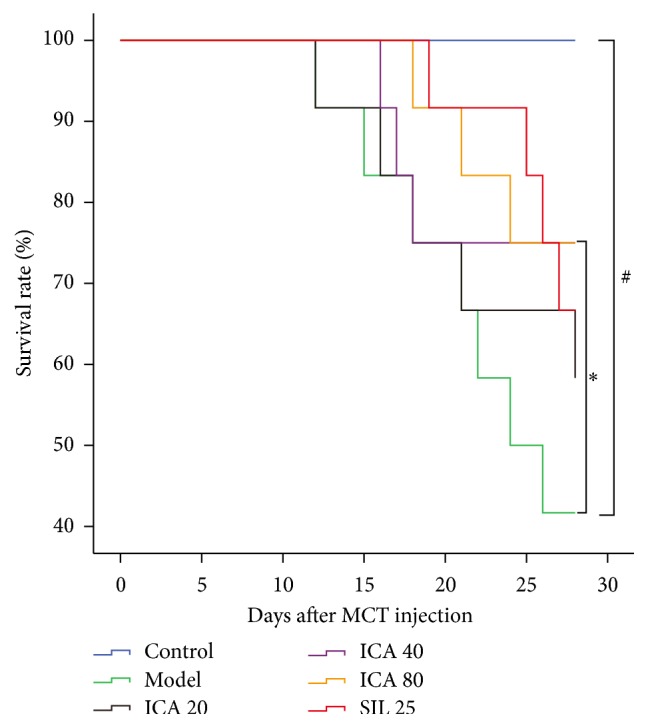
Effect of ICA on survival rates. Dose: mg/kg/d. Mortality was observed daily and the figure shows the survival rate at each time point, setting the survival rate at the start of model established as 100%. In order to easily read, the survival rates data of L-arg treatment groups, L-arg (200 mg/kg/d) with L-NAME (20 mg/kg/d) group, and ICA (40 mg/kg/d) with L-NAME (20 mg/kg/d) group are not presented in Figure 1. Compared with control group ^#^ *p* < 0.05, compared with model group ^*∗*^ *p* < 0.05, Log-Rank test.

The effects of ICA on mPAP and RVHI are shown in Figures 1 2 and 3. The mPAP value in model group was 53.5 ± 6.7 mmHg, showing a significant elevation compared with the control group (16.2 ± 3.2 mmH) (*p* < 0.05), and RVHI also increased from 16.2 ± 3.1% in the control group to 32.8 ± 3.8% in the model group (*p* < 0.05). Sildenafil (25 mg/kg/d), a PDE-5 inhibitor, conspicuously decreased the mPAP (26.2 ± 8.5 mmHg) and ameliorated right ventricle hypertrophy (RVHI 25.9%) (*p* < 0.05). The data have shown that PH model was successfully established and the test system was reliable. Administration of ICA (20, 40, and 80 mg/kg/d) suppressed the mPAP in a dose-dependent manner. L-arg (200 mg/kg/d) treatment had a similar effect on mPAP. It was notable that L-NAME (20 mg/kg/d), a NOS inhibitor, could significantly inhibit the effect of ICA (40 mg/kg/d) and L-arg on mPAP (*p* < 0.05). The inhibitory effects of ICA and L-arg on RVHI were similar to that of mPAP in a consistent manner, and L-NAME had a tendency to inhibit both ICA (40 mg/kg/d) and L-arg effects on RVHI, but there was no statistical difference (*p* > 0.05).

### 3.3. ICA Treatment Suppresses Lung Vascular Remodeling Induced by MCT in Rats

**Figure 4:**
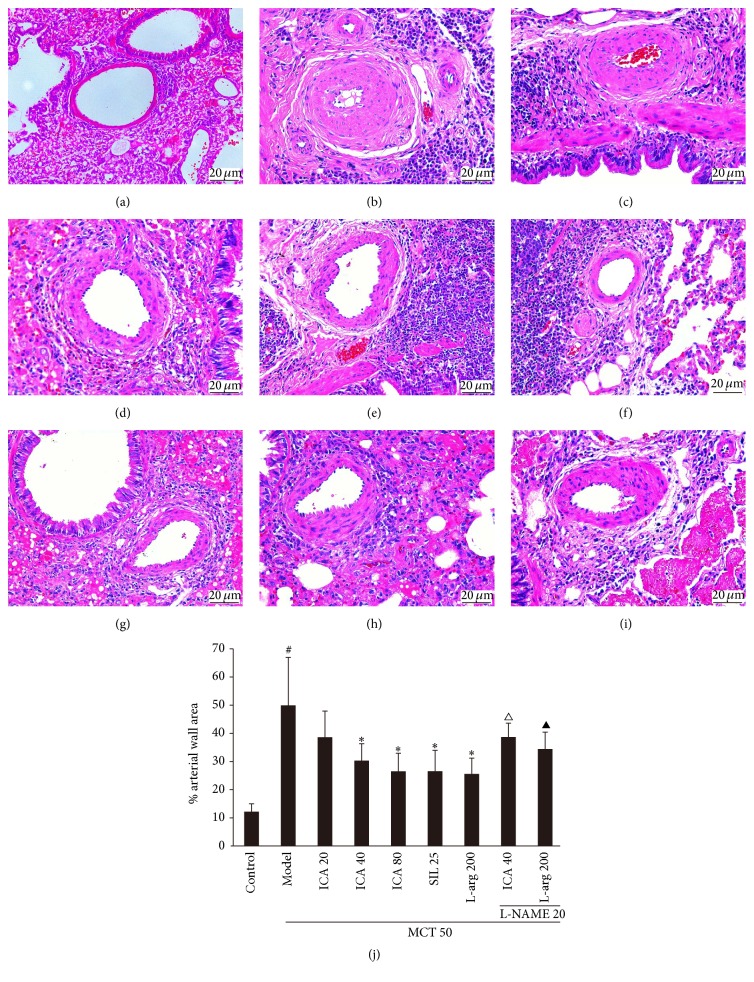
Effect of ICA on lung vascular remodeling induced by MCT in rats (mean ± SD, *n* = 5–12). Dose: mg/kg/d. (a)~(i) Representative images of H&E staining lung sections from every group rats. (j) Bar graph of % pulmonary artery wall area (diameter: 50 to 100 *μ*m). Groups: (a) control; (b) model; (c) ICA (20 mg/kg/d); (d) ICA (40 mg/kg/d); (e) ICA (80 mg/kg/d); (f) SIL (25 mg/kg/d); (g) L-arg (200 mg/kg/d); (h) ICA (40 mg/kg/d) combining with L-arg (200 mg/kg/d); (i) L-NAME combining with L-arg (200 mg/kg/d). Compared with control group ^#^ *p* < 0.05; compared with model group ^*∗*^ *p* < 0.05; compared with ICA (40 mg/kg/d) group ^△^ *p* < 0.05; compared with L-arg (200 mg/kg/d) group ^▲^ *p* < 0.05.

The normal histomorphology of pulmonary artery was shown in Figure 4(a). It was shown that MCT injection led to a conspicuous artery remodeling, which was characterized by intimal hyperplasia and medial hypertrophy and vascular lumen stenosis in the distal arterioles (Figure 4(b)). ICA-treatment could alleviate the artery remodeling (Figures 4(c), 4(d), and 4(e)) as well as sildenafil and L-arg (Figures 4(f) and 4(g)); the intimal hyperplasia was improved more significantly than medial hypertrophy. When the protective effect of ICA on MCT-induced pulmonary vascular remodeling was analyzed quantitatively by calculating the percentage of the artery wall area and its cross-sectional area, it was found that MCT treatment evoked an increase in percentage to approximately 4 times, administration of ICA significantly decreased the elevated percentage (*p* < 0.05), and it was the same in sildenafil and L-arg groups (*p* < 0.05). L-NAME markedly inhibited protective function of ICA (40 mg/kg/d) and L-arg (*p* < 0.05) on MCT-induced pulmonary vascular remodeling (Figure 4(j)), similar to its influence on the effects of ICA on mPAP and RVHI.

### 3.4. ICA Administration Ameliorates Lung Function in MCT-PH Model Rats

**Figure 5:**
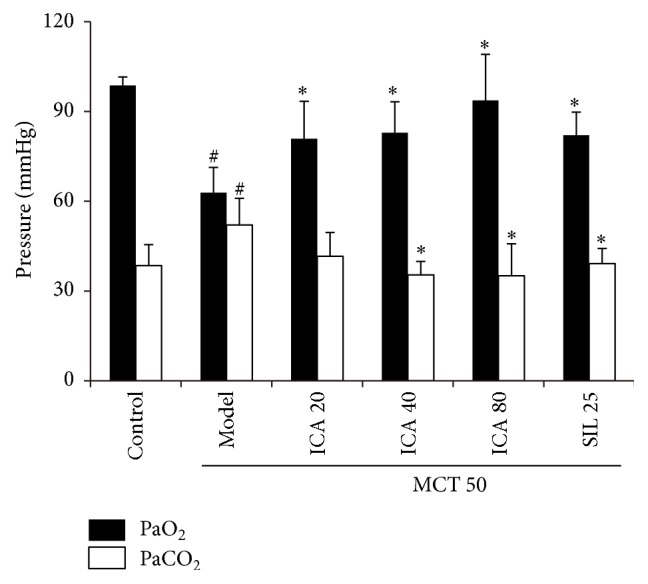
Effect of ICA on arterial blood partial pressure of oxygen and carbon dioxide (mean ± SD, *n* = 5–12). Dose: mg/kg/d. PaO_2_: arterial blood partial pressure of oxygen; PaCO_2_: arterial blood partial pressure of carbon dioxide. Compared with control group ^#^ *p* < 0.05; compared with model group ^*∗*^ *p* < 0.05.

In the present study, the partial pressures of arterial blood oxygen and carbon dioxide, a pair of very important indices reflecting the pulmonary function, were checked. It was found that, compared with the control, the arterial blood partial pressure of oxygen significantly decreased (98.7 ± 2.4 mmHg to 62.9 ± 8.5 mmHg) with the retention of carbon dioxide in model group. Administration of ICA could reverse the deterioration of pulmonary function resulting from MCT injection (*p* < 0.05) (Figure 5).

### 3.5. ICA Enhances Function of NO/cGMP Signaling Pathway in MCT-PH Model Rats

**Figure 6:**
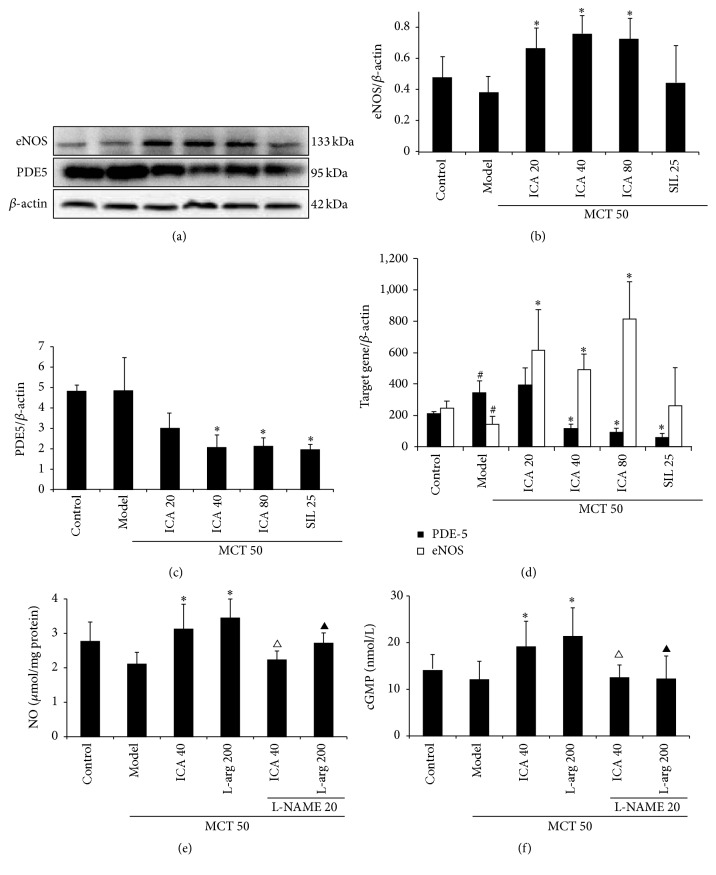
ICA enhances function of NO/cGMP signaling pathway in MCT-PH model rats (mean ± SD, *n* = 5–12). Dose: mg/kg/d. (a) Representative Western blots from lung homogenate; (b) quantitative bar graph of eNOS expression of lung tissue; (c) quantitative bar graph of PDE5 expression of lung tissue. (d) Bar graph of eNOS and PDE5 mRNA expression; (e) bar graph of NO content of lung tissue; (f) bar graph of cGMP content of lung tissue; compared with control group ^#^ *p* < 0.05; compared with model group ^*∗*^ *p* < 0.05; compared with ICA (40 mg/kg/d) group ^△^ *p* < 0.05; compared with L-arg (200 mg/kg/d) group ^▲^ *p* < 0.05.

For investigating whether protective effects of ICA on PH induced by MCT were associated with NO/cGMP signaling pathway, mRNA and protein expression of eNOS and PDE5 were investigated, although protein expression of both eNOS and PDE5 had no notable difference between control group and model group (*p* > 0.05), ICA (20, 40, and 80 mg/kg/d) administrations caused a significant difference in protein expression of eNOS (*p* < 0.05), and ICA (40, 80 mg/kg/d) and sildenafil (25 mg/kg/d) treatment decrease the protein expression of PDE5 (Figures 6(a), 6(b), and 6(c)) (*p* < 0.05). The result of Real-Time RT-PCR indicates that ICA upregulates expression of eNOS mRNA and downregulates expression of PDE5 mRNA compared with model group (Figure 6(d)). For further confirming effect of ICA on NO/cGMP signaling pathway, the content of NO and cGMP in lung tissue was measured. Figures 6(e) and 6(f) showed that administration of ICA (40 mg/kg/d) or L-arg (200 mg/kg/d) could significantly increase the contents of NO and cGMP in lung tissue versus model group (*p* < 0.05). Treatment of L-NAME (20 mg/kg/d), a NOS inhibitor, could abolish the effects of ICA and L-arg on NO and cGMP contents (*p* < 0.05).

## 4. Discussion

Although the causes and pathogenesis of PH have still not been fully elucidated, the understanding of PH in the recent three decades has made the rapid progress in pathobiological processes and therapeutic targets, which partly benefited from development of PH animal models [22]. The classical rat MCT-PH model was introduced more than 50 years ago [23]. MCT is metabolized by hepatic cytochrome P450 3A and changed to monocrotaline pyrrole, an active form, which injures pulmonary vascular endothelium and causes PH [24], which is characterized by gradually increasing pulmonary arterial pressures and secondary vascular remodeling in 1~2 weeks after MCT single subcutaneous injection [25]. The typical histological changes present in MCT model include intimal hyperplasia, medial hypertrophy, adventitial proliferation/fibrosis, and occlusion of small arteries [26]. With development of the disease, the impairment of heart and lung structures occurs and finally leads to heart failure and respiratory failure [22]. Our study found that, at the end of the 4th week after MCT injection, a series of pathological changes in rats could be detected, including elevated mPAP, pulmonary artery remodeling, right ventricle hypertrophy, and deteriorated respiratory exchange function, with a lower survival rate. These findings suggested that the model was successfully established.

In the present study, the effects of ICA on PH progression and survival benefit were investigated in MCT-induced PH rats, employing the sildenafil as a positive control. ICA was given by intragastric administration for a period from the 8th day to 28th day after MCT injection, which is the aggressive phase of progression of MCT-PH model and is usually chosen as the therapeutic time window by some investigators. To our attention, ICA 20 mg/kg/d significantly suppressed the increases in mPAP, whereas suppressing effects of ICA 20 mg/kg/d on RVHI and % arterial wall area were not complete in our experiment, suggesting that ICA 20 mg/kg/d dosage was close to minimum effective dose of attenuating MCT-induced PH in rats. Enhancement of reactivity of pulmonary artery to vasoconstrictive substances and pulmonary artery remodeling caused by monocrotaline pyrrole toxicity result in increase of mPAP and compensatory hypertrophy of the right ventricle due to overload in MCT-PH rat model [7], ICA treatment could decrease mPAP in a dose-dependent fashion at doses of 20, 40, and 80 mg/kg/d, and however ameliorated effect of ICA on pulmonary artery remodeling was not significant compared with model group at doses of 20 mg/kg/d level, suggesting that decrease in mPAP in ICA-treated group did not fully result from attenuation of vascular remodeling. On the other hand, degree of right ventricle hypertrophy was notably ameliorated in ICA-treated group due to shrinking of afterload. ICA treatment also significantly extended survival time in dose-dependent fashion with statistical difference at the highest dose. These results strongly suggested that ICA is an effective reagent of anti-PH induced by MCT.

With the increased understanding of the pathogenesis of PH, many investigators have focused on NO/cGMP signaling pathway as the target of new drug development. In this study, in spite of NO and cGMP content, protein expression of eNOS and PDE5 in lung tissue had no notable statistical difference except expression of eNOS mRNA downregulation and expression of PDE5 mRNA upregulation in the MCT-treated group in comparison to that in the control group. We found that ICA administration could upregulate the expression of eNOS gene at 20, 40, and 80 mg/kg/d dose levels and downregulated the expression of PDE5 gene at 40 and 80 mg/kg/d dose levels in lung tissue of the MCT-injection rats. Further research found that the contents of NO of lung tissue homogenate were significantly increased by ICA with the dose level of 40 mg/kg/d, which coincided with expression changes in the eNOS, and the contents of cGMP were also elevated by ICA with the same dose level that resulted from increasing of NO contents and downregulated the expression of PDE5 protein. For further confirmation of whether or not the anti-PH effect of ICA related to its enhancement of NO/cGMP pathway, we used L-NAME, a NOS inhibitor, to interfere with the effects of ICA on the NO/cGMP pathway, and L-arg was taken in this experiment protocol as a positive control. The results showed that L-NAME could inhibit anti-PH effect of ICA: abolishing the decreasing effect of ICA and L-arg on mPAP, attenuating amelioration of ICA and L-arg on artery remodeling, and abolishing the enhancing effects of ICA and L-arg on NO and cGMP contents. It is similar to the influence of L-NAME on L-arg anti-PH effect. L-NAME-treatment could have tendencies to inhibit the decreasing effect of ICA and L-arg on right ventricle hypertrophy, although there was no statistical difference. From the results, it was very clearly suggested that ICA enhanced the NO/cGMP signal pathway in the lung tissue. Therefore, we believe that the anti-PH effect of ICA is involved in the enhancement of the NO/cGMP pathway.

## 5. Conclusions

In conclusion, ICA is effective in protecting against MCT-induced PH in rats; the mechanism for its anti-PH may be involved in the enhancement of the NO/cGMP signaling pathway.

## Figures and Tables

**Figure 2:**
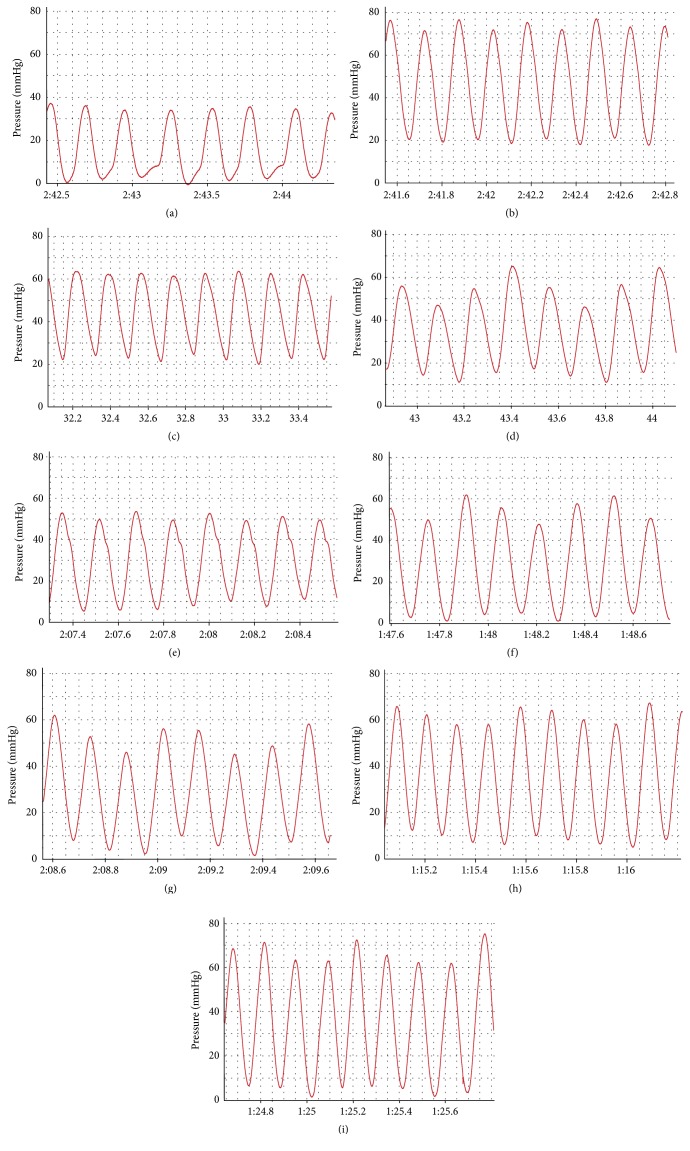
Representative pulmonary arterial pressure curve. Groups: (a) control; (b) model; (c) ICA (20 mg/kg/d); (d) ICA (40 mg/kg/d); (e) ICA (80 mg/kg/d); (f) SIL (25 mg/kg/d); (g) L-arg (200 mg/kg/d); (h) ICA (40 mg/kg/d) combining with L-arg (200 mg/kg/d); (i) L-NAME combining with L-arg (200 mg/kg/d).

**Figure 3:**
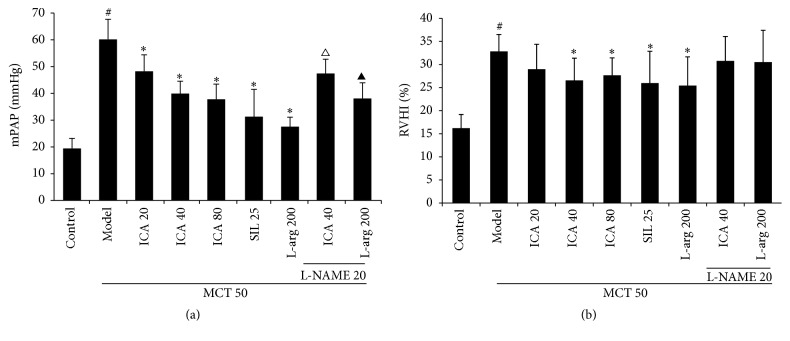
Effects of ICA treatment on mPAP (a) and RVHI (b) (mean ± SD, *n* = 5–12). Dose: mg/kg/d. mPAP: mean pulmonary artery pressure; RVHI: right ventricular hypertrophy index. Compared with control group ^#^ *p* < 0.01; compared with model group ^*∗*^ *p* < 0.05; compared with ICA (40 mg/kg/d) group ^△^ *p* < 0.05; compared with L-arg (200 mg/kg/d) group ^▲^ *p* < 0.05.
